# Vaginal Treatment of Vaginal Cuff Dehiscence with Visceral Loop Prolapse: A New Challenge in Reparative Vaginal Surgery?

**DOI:** 10.1155/2014/257398

**Published:** 2014-11-24

**Authors:** Salvatore Andrea Mastrolia, Edoardo Di Naro, Luca Maria Schonauer, Maria Teresa Loverro, Beatrice Indellicati, Mario Barnaba, Giuseppe Loverro

**Affiliations:** Department of Obstetrics and Gynecology, School of Medicine, University Hospital Policlinico of Bari and University of Bari “Aldo Moro”, Piazza Giulio Cesare 11, 70124 Bari, Italy

## Abstract

Vaginal cuff dehiscence is a rare, but potentially morbid, complication of total hysterectomy and refers to separation of the vaginal cuff closure. The term vaginal cuff dehiscence is frequently interchanged with the terms of cuff separation or cuff rupture. All denote the separation of a vaginal incision that was previously closed at time of total hysterectomy. After dehiscence of the vaginal cuff, abdominal or pelvic contents may prolapse through the vaginal opening. Bowel evisceration, outside the vulvar introitus, can lead to serious sequelae, including peritonitis, bowel injury and necrosis, or sepsis. Therefore, although prompt surgical and medical intervention is required to replace prolapsed structures, the main problem remains the reconstruction of vaginal vault. In case of recent hysterectomy, vaginal reparation only requires the approximation of vaginal walls, including their fascia, while if dehiscence occurs after a long time from hysterectomy, the adequate suspension of the vaginal vault has to be taken into consideration. In this report we describe the case of a postmenopausal patient, undergoing surgical emergency because of the evisceration of an intestinal loop through a dehiscence of vaginal vault, after numerous reconstructive vaginal surgeries for vaginal prolapse. This paper analyzes clinical circumstances, risk factors, comorbidity, and clinical and surgical management of this complication.

## 1. Introduction

Vaginal cuff dehiscence is a rare, but potentially morbid, complication of total hysterectomy and refers to separation of the vaginal cuff closure. The term vaginal cuff dehiscence is frequently interchanged with the terms of cuff separation or cuff rupture. All denote the separation of a vaginal incision that was previously closed at time of total hysterectomy.

After dehiscence of the vaginal cuff, abdominal or pelvic contents may prolapse through the vaginal opening. Bowel evisceration, outside the vulvar introitus, can lead to serious sequelae, including peritonitis, bowel injury and necrosis, or sepsis.

Therefore, although prompt surgical and medical intervention is required to replace prolapsed structures, the main problem is the reconstruction of vaginal vault.

In case of recent hysterectomy, vaginal reparation only requires the approximation of vaginal walls, including their fascia, while if dehiscence occurs after a long time from hysterectomy, the adequate suspension of the vaginal vault has to be taken into consideration.

In this report, we describe the case of a postmenopausal patient, presenting evisceration of an intestinal loop through a dehiscence of vaginal vault, after numerous reconstructive vaginal surgeries for vaginal prolapse. This paper analyzes clinical circumstances, risk factors, comorbidity, and clinical and surgical management for this complication.

## 2. Case Report

A 58-year-old patient, gravida 1 para 1, presented at Emergency Service of the Department of Obstetrics and Gynecology, University Hospital of Bari, for a vaginal herniation of a bowel loop outside the vulvar introitus.

Past gynecological history revealed, ten years before admission, vaginal hysterectomy following diagnosis of uterine prolapse and uterine fibromatosis. After three years, the patient had a vaginal anterior prolapse and underwent anterior vaginoplasty; lastly, five years after hysterectomy she underwent promontory sacropexy for vaginal vault prolapse, with mesh implant removed within three years following erosion and infection. All the surgeries were uneventful and were not followed by any postoperative complication.

Noteworthy, two months before vaginal cuff dehiscence, she started a treatment with steroids (metilprednisolone 8 mg) following a not well-specified diagnosis of connective tissue disorder. Her body mass index was 25.71 kg/m^3^.

**Figure 1:**
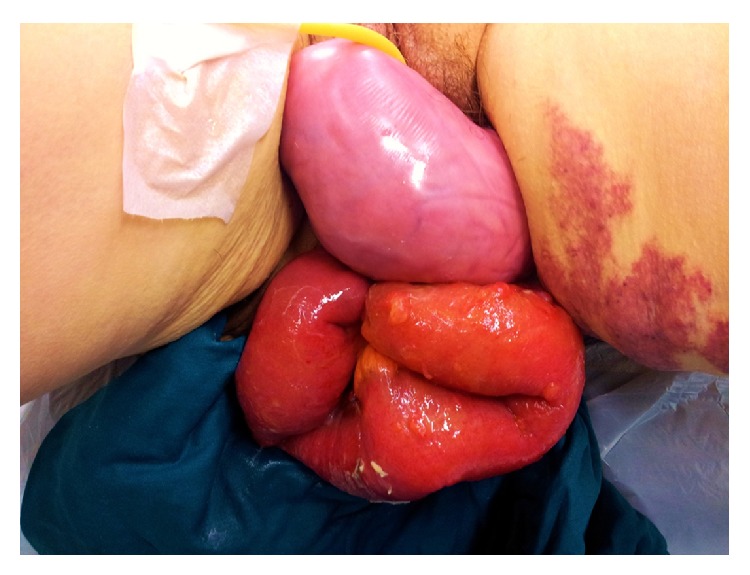
Small bowel loops prolapsing through a dehiscence of the vaginal vault.

At admission, physical examination evidenced the presence of a small bowel loop with the length of about 15 cm through the vulvar edge, associated with a large prolapsed anterior vaginal wall (Figure 1).

The bowel loop herniated through a dehiscence of about 3 cm located on the posterior vaginal wall, appeared tense, edematous, and cyanotic, with signs of vascular compromising and covered by fibrinous tissue. Moreover, after consultation with a general surgeon, a bowel resection was not considered necessary. The abdomen was treatable, not painful, with medium thickness, and moderate meteorism.

Laboratory-instrumental evaluation showed no signs of hydroelectrolytic and hemodynamic failure. The ECG showed a sinus rhythm at 66 bpm.

The herniated loop was immediately protected with sterile towels and disinfection of the surrounding surfaces.

Then, the surgical approach was divided into two stages. The first step was to perform a suprapubic transverse laparotomy to replace the eviscerated intestinal loops in their natural site, after confirming their integrity. The abdominal procedure was ended with Mc call culdoplasty.

Then, a vaginal procedure consisted in a longitudinal incision of the vaginal wall. In the site of dehiscence, the vaginal wall appeared thin, fibrotic and with a 5 cm area of necrosis so it was not useful for repair because it was not vascular. Therefore, after removal of this necrotic nonvascular part, pubocervical fascia was closed and elevator ani and pubococcygeal muscles were approximated on the medium line detached suture and vaginal walls were sutured by interrupted delayed absorbable monofilament (the same method was used for cuff closure after hysterectomy and mesh removal) and anchored to elevator ani muscles, remaining a vaginal length of 4 cm.

The patient was discharged on the fourth day after surgery, with a clinical and surgical completely stabilized condition. Clinical control, at one year time after surgery, revealed a 4 cm vaginal length, still firmly attached to elevator ani muscle, impeding dyspareunia sexual intercourses.

## 3. Discussion

Historically, vaginal vault dehiscence, either with or without associated intestinal evisceration, is considered one of the possible surgical complications of vaginal hysterectomy, although it can occur after abdominal hysterectomy.

In both circumstances, we are faced with a defect of the reconstruction of the various layers, often in the context of an already present tissue weakness due to prior surgery or inadequate vaginal suture technique.

This is a rare but serious complication and can be defined as a partial or full thickness separation of the anterior and posterior edges of the vaginal cuff with or without intestinal evisceration [1].

Its exact incidence is difficult to establish since it is not well documented in the literature. This is mainly due to its not fully clarified pathogenesis and the fact that literature epidemiological rates are affected by the extreme heterogeneity of studies developed in the last years. Anyway, there are often conflicting results among the different reports [2–8].

Literature has analyzed this condition in the last twenty years, with particular attention to the incidence of this complication in relation to different surgical techniques. It seems that risk would be increased by laparoscopic surgery instead of laparotomic procedure or vaginal hysterectomy. In this sense, the incidence rate varies between 0.14% and 0.27% considering the totality of pelvic surgical approaches and between 1% and 4.1% considering only robotic hysterectomy and total laparoscopic hysterectomy [9–14].

The prolapse of bowel loops through the vagina, however, can be associated with repeated interventions of suspension or vaginal correction. In these circumstances, a tissue sclerosis can be determined and along with the weakening of the suspension system and postmenopausal atrophy predispose to dehiscence of vaginal vault scarring area.

Emblematic of our case was the presence of multiple factors predisposing to inflammation, sclerosis, loss of vascularization, and atrophy of the support systems up to result in a spontaneous rupture of the prolapsed vaginal vault.

Somkuti et al. [15] examined 10 risk factors for prolapsing of the vaginal vault following abdominal or vaginal hysterectomy: (1) poor technique, (2) postoperative infection, (3) occurrence of hematoma, (4) sexual intercourse before complete healing, (5) age, (6) postsurgical radiotherapy, (7) use of corticosteroids, (8) trauma or rape, (9) previous vaginoplasty, and (10) Valsalva maneuver.

Clearly, we have to consider vaginal atrophy an additional and serious risk factor not only due to estrogenic postmenopausal drop, but also related to collagen diseases, hypothyroidism, smoke, and corticosteroids, as in our case [16–18].

Probably, a causal factor expected to grow in the near future and present in our case is repetitive vaginal surgery for prolapse, associated with placement of prolene mesh, removed for inflammation. Repeated surgery promotes progressive sclerosis of vaginal fascia, and the presence of inflammatory for foreign body can contribute to the event, which undoubtedly in our case was promoted by the recent administration of cortisone in large doses.

Of interest, we have to consider that statistic increase of prolapse of vaginal vault associated with the possibility for all the risk factor to act for a number of decades as suggested by the increase in life expectancy.

At the moment, there is no consensus on the ideal method of surgical repair in case of dehiscence of the vaginal vault.

A review published in April 2012 by the American Journal of Obstetrics and Gynecology [19] reviewed a total of 73 cases of surgical repair of dehiscent vaginal vault with evisceration of intestinal loops.

Case reports, case series, and retrospective cohort studies were taken into account. The resulting data revealed that 51% of dehiscence was repaired vaginally, 32% following the abdominal approach, 2% laparoscopic, and 10% through a combined approach (abdominal and vaginal or laparoscopic and vaginal), while in 5% of cases a secondary intention healing was preferred. Looking at these data, an infrequent use of laparoscopy can be noted. This can be the result of a reduced availability, in the past, of surgeons with expertise in laparoscopy. Moreover, the increase in laparoscopic skills for this kind of surgeries is leading to more and more reports in literature, raising a question regarding if a new method should be discussed in the approach to the above mentioned complication of hysterectomy.

Until the date, no method appears to be preferable in absolute terms with respect to another. The choice must necessarily be based on a number of variables, such as the clinical performance of the patient, the surgeon's experience, the possible ischemic and/or mechanical damage occurring to abdominal organs, the presence or absence of intestinal evisceration associated with the possibility to visualize and repair the vaginal mucosa in an appropriate manner, and the ability to perform additional necessary procedures [19].

In our case, a combined vaginal and abdominal repair appeared the most reassuring, especially given the previous surgical history of the patient.

The technique of culdoplasty following McCall avoids the dissection of the hernial sac or the peritoneal excess which, moreover and as in our case, can also be removed subsequently by the vaginal route, if exuberant and necrotic. The high median approach to uterosacral ligaments with high closure of the peritoneum is associated with a 6.1% risk of recurrence of prolapse with respect to 30.3% of the simple approach of the peritoneum of Douglas according to the technique of Moschcowitz.

Our intervention can be considered similar to colpectomy following Percy-Perl procedure. This was performed in a partial way in consideration of the need to eliminate the entire vaginal exuberant wall, part of which with necrotic aspects and fibrosis.

## 4. Conclusion

No method appears to be preferable in absolute terms for the repair of vaginal cuff dehiscence. The choice must necessarily be based on a number of variables.

Undoubtedly, the increase in life expectancy and of vaginal reparative procedures and the increasing use of prosthetic material can increase the risk of occurrence of these events and put us in front of the need to verify the need of adopting new therapeutic strategies.

Reporting these results, although in the form of individual cases, may be useful to understand the pathophysiology and to find prevention procedures, considering the conflicting information on the subject, reported in literature [2–8].

In the near future, new criteria should be adopted in surgical vaginal dehiscence repair, taking into account the anatomical situation and the needs of the patient, but especially considering that this kind of complication can present itself as emergent situations, without availability of surgeons with expertise in pelvic reparative surgery.
